# Randomized clinical trial of prophylactic transanal irrigation *versus* supportive therapy to prevent symptoms of low anterior resection syndrome after rectal resection

**DOI:** 10.1002/bjs5.50160

**Published:** 2019-03-18

**Authors:** H. R. Rosen, W. Kneist, A. Fürst, G. Krämer, J. Hebenstreit, J. F. Schiemer

**Affiliations:** ^1^ Faculty of Surgical Oncology, Sigmund Freud University Vienna Austria; ^2^ Department of Surgery, Hospital St John of God Graz Austria; ^3^ Department of General, Visceral and Transplantation Surgery, Johannes Gutenberg University Mainz Germany; ^4^ Department of Surgery, Caritas‐Hospital St Josef Regensburg Germany

## Abstract

**Background:**

Low anterior resection syndrome (LARS) is a frequent problem after rectal resection. Transanal irrigation (TAI) has been suggested as an effective treatment in patients who have developed LARS. This prospective RCT was undertaken to evaluate the effect of TAI as a prophylactic treatment to prevent symptoms of LARS.

**Methods:**

Patients who had undergone ultralow rectal resection were randomized to start TAI on a daily basis, or to serve as a control with supportive therapy only after ileostomy closure. All patients were seen after 1 week, 1 month and 3 months, and the maximum number of defaecation episodes per day and night documented during follow‐up. Wexner score, LARS score and Short Form 36 questionnaire responses were evaluated in both groups.

**Results:**

Thirty‐seven patients could be evaluated according to protocol (TAI 18, control 19). The maximum number of stool episodes per day and per night was significantly lower among patients who underwent TAI at 1 month (median 3 *versus* 7 episodes/day in TAI *versus* control group, *P* = 0·003; 0 *versus* 3 episodes/night, *P* = 0·001) and 3 months (3 *versus* 5 episodes per day, *P* = 0·006; 0 *versus* 1 episodes/night, *P* = 0·002). LARS scores were significantly better in the TAI group after 1 month (median 16 *versus* 32 in control group; *P* = 0·044) and 3 months (9 *versus* 31; *P* = 0·001). A significantly better result in terms of Wexner score was seen in the TAI group after 3 months (median 2 *versus* 6 in controls; *P* = 0·046).

**Conclusion:**

Prophylactic TAI led to a significantly better functional outcome compared with supportive therapy for up to 3 months. Registration number: DRKS00011752 ( http://apps.who.int/trialsearch/).

## Introduction

Preservation of the anal sphincter during rectal resection can be regarded as one of the historical milestones in colorectal surgery^1^, ^2^. Coupled with increasing knowledge regarding local and lymphatic spread, a level of resection with concomitant sphincter preservation can be achieved in patients with ultralow rectal cancer down to 2 cm from the dentate line^2^, ^3^, ^4^. Ultralow and intersphincteric rectal resection with coloanal anastomosis together with neoadjuvant chemotherapy/radiotherapy have led to excellent oncological results, with avoidance of a permanent stoma^5^.

Preservation of the anal sphincter, however, has not been able to guarantee an excellent functional outcome. Frequent bowel movements, stool fragmentation, defaecatory urgency and incontinence have been reported in up to 80 per cent of patients following rectal resection^6^, ^7^, ^8^, ^9^. These symptoms are summarized under the term low anterior resection syndrome (LARS). Research into this condition has led to the development of a standardized and validated scoring system (LARS score), providing a tool to evaluate potential treatments for patients affected by this disorder^10^, ^11^.

Transanal irrigation (TAI) has been shown to be effective in improving function in patients suffering from LARS for sustained lengths of time^12^, ^13^. The aim of this multicentre randomized trial was to evaluate the effect of immediate prophylactic application of TAI after closure of the protective ileostomy after low rectal resection.

## Methods

This was a stratified (according to centre and formation of a neoreservoir or straight coloanal anastomosis) RCT performed at three institutions in Germany and Austria.

Inclusion criteria for participation were: patients who had undergone rectal resection for rectal cancer (with or without pouch reconstruction) with an anastomotic height less than 5 cm above the dentate line measured by rigid proctoscopy; proof of complete healing of the anastomosis by endoscopy or radiology before stoma closure; informed patient consent; and mental and physical capability of the patient to perform TAI.

The primary endpoint of the study was the maximum number of defaecation episodes during daytime at 1 month after ileostomy closure. Secondary endpoints were the maximum number of defaecation episodes per night, and effect on quality of life (QoL) measured by LARS score^11^, Wexner incontinence score^14^, and the mental and physical components of the Short Form 36 questionnaire (SF‐36®; Optum, Eden Prairie, Minnesota, USA)^15^.

Patients who fulfilled the inclusion criteria were randomized on the day before ileostomy closure. Presence of a straight coloanal anastomosis *versus* construction of a reservoir (J pouch, side‐to‐end anastomosis or coloplasty) and centre location were used as stratification criteria during the randomization process. Once eligibility for participation had been confirmed, randomization was done at an independent centre (not belonging to 1 of the participating surgical units) via an online process on the day before ileostomy closure. An open‐source customizable minimization program (MinimPy; http://minimpy.sourceforge.net) was used for allocation of patients, and to minimize possible imbalances for the factors centre and neoreservoir *versus* straight coloanal anastomosis.

Patients randomized to the TAI group received intensive counselling and training in use of the Peristeen® device (Coloplast, Humlebaek, Denmark), and started the first irrigation under the guidance of a specially trained stoma/incontinence therapist once passage of the first stool had been documented. According to protocol, irrigation was performed with 1000 ml tap water every 24 h. Patients in the control group received best supportive therapy according to the individual treatment protocols available at each participating centre. These protocols consisted of dietary (bulk forming) modifications, biofeedback‐assisted pelvic floor training for patients who reported episodes of incontinence, and treatment with loperamide.

Follow‐up was planned at the end of the first week, and first and third months after stoma closure. Patients were instructed to document the number of defaecation episodes (visits to the toilet for defaecation) during the daytime as well as at night using a daily diary. The Wexner (incontinence) score^14^ was documented along with the total time on the toilet needed to empty the irrigation volume (1000 ml).

QoL was evaluated using the LARS score and the SF‐36® questionnaire. Responses to the SF‐36® questionnaire were used to calculate the mental and physical component scores during follow‐up.

The study was performed in accordance with the Declaration of Helsinki, Good Clinical Practice, and was reviewed and approved by the local ethics committee at each participating centre.

### Statistical analysis

In an earlier joint Austrian–Swiss study, the authors had been able to reduce the median number of defaecation episodes in patients with LARS from a median of 8 to 1 during the day, and from 3 to 0 at night using TAI every 24 h^12^. Based on these observations, and setting a minimum power of 0·80 and a significance level of 0·05 for a two‐sided hypothesis, a minimum of 18 patients per group was deemed an acceptable sample size.

Continuous data are presented as median (range), and the Mann–Whitney *U* test was used to compare the groups. Categorical variables were evaluated by use of the χ^2^ test. *P* < 0·050 was taken as the level of statistical significance.

## Results

**Figure 1:**
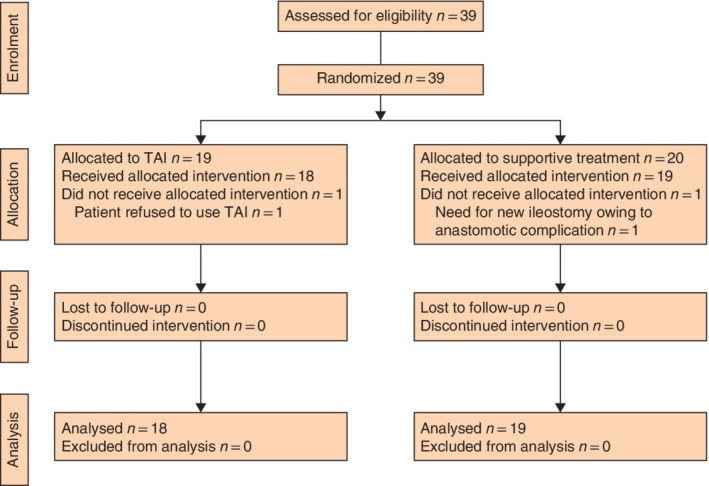
CONSORT diagram for the trial. TAI, transanal irrigation

In total, 39 patients were allocated between February 2016 and April 2018. One patient randomized to TAI refused to continue with irrigation after 1 month of follow‐up. One patient allocated to the control group experienced a surgical complication that required a new ileostomy, leaving 37 patients treated according to the protocol (*Fig*. 1). Patient characteristics are summarized in *Table* 1. As sex was not a stratification criterion during the randomization process, the sex distribution differed significantly between the two groups (*P* = 0·015).

**Table 1:** Patient characteristics

	TAI (*n* = 18)	Control (*n* = 19)
Age (years)*	58·5 (52–70)	58 (42–80)
Sex ratio (M : F)	12 : 6	5 : 14
Height of anastomosis above dentate line (cm)*	3 (2–5)	3·5 (2–5)
Preoperative radiation	15	14
Reconstruction type		
Pouch Straight anastomosis	6 12	4 15

* Values are median (range). TAI, transanal irrigation.

In total, 29 patients received neoadjuvant radiotherapy (48–52 Gy over 5 weeks). There was no significant difference in the number of patients receiving radiotherapy between TAI and control groups (*P* = 0·482).

In four patients randomized to TAI, the coloanal anastomosis was regarded as appropriate for ileostomy closure but too narrow to allow safe insertion of the irrigator. According to protocol, these patients were instructed to irrigate by use of a 28‐Fr Foley catheter and 100‐ml syringes, so that irrigation with 1000 ml water was feasible. Patients received daily training sessions in the first week while still in hospital after ileostomy closure, followed by appointments with the stoma/incontinence therapist on an outpatient basis (as requested by the patient), resulting in no technical problems or complications related to TAI.

Results of evaluation of the primary endpoint (number of defaecation episodes during the daytime after 1 month) along with other parameters investigated are shown in *Table* 2. In the first week after ileostomy closure, patients in the TAI group had more defaecation episodes in the daytime than those in the control group. The number of defaecation episodes during the day was significantly lower in the TAI group than in the control group at 1 and 3 months after ileostomy closure. Although the number of defaecation episodes during the night did not differ significantly between the two groups after 1 week, patients in the TAI group also reported significantly fewer bowel movements during the night at 1 and 3 months.

**Table 2:** Results at follow‐up

	TAI	Control	*P* *
1 week			
Maximum no. of defaecations/day	10 (3–34)	4 (2–20)	0·004
Maximum no. of defaecations/night	3 (0–8)	2 (2–20)	0·757
Wexner score	7·5 (0–20)	10 (0–20)	0·238
SF‐36® mental component	48 (29–57)	55 (29–63)	0·543
SF‐36® physical component	42 (19–54)	34·5 (29–58)	0·965
LARS score	37·5 (4–42)	32 (3–41)	0·177
1 month			
Maximum no. of defaecations/day	3 (1–10)	7 (3–30)	0·003
Maximum no. of defaecations/night	0 (0–6)	3 (0–6)	0·001
Wexner score	4 (0–17)	10 (0–17)	0·087
SF‐36® mental component	51 (28–59)	55 (29–60)	0·195
SF‐36® physical component	44 (35–55)	49 (20–58)	0·356
LARS score	16 (4–39)	32 (2–41)	0·044
3 months			
Maximum no. of defaecations/day	3 (1–10)	5 (3–12)	0·006
Maximum no. of defaecations/night	0 (0–2)	1 (1–5)	0·002
Wexner score	2 (0–11)	6 (0–17)	0·046
SF‐36® mental component	55 (31–60)	57 (26–63)	0·436
SF‐36® physical component	50 (39–64)	51 (37–61)	0·741
LARS score	9 (0–34)	31 (3–42)	0·001

Values are median (range). TAI, transanal irrigation; SF, Short Form; LARS, low anterior resection syndrome.

* Mann–Whitney *U* test.

The median maximum time on the toilet to empty the irrigation volume was 47 (22–70) min at 1‐week, 44 (30–65) min at 1‐month and 45 (30–60) min at 3‐month follow‐up.

Wexner incontinence scores were lower in the TAI group during follow‐up, but statistical significance was reached only at the last follow‐up 3 months after ileostomy closure (*Table* 2).

With regard to the effect of TAI on the LARS score, no significant difference between the two groups was observed 1 week after stoma closure. However, after 1 and 3 months, patients in the TAI group showed significantly better results of LARS evaluation compared with controls (*Table* 2). In contrast, analysis of the mental and physical components of the SF‐36® questionnaire did not reveal any difference between the groups at any time point.

Because the sex distribution differed significantly between the two groups, a further analysis of all variables investigated was undertaken with sex as a stratification criterion; this had no impact on the results (data not shown).

## Discussion

TAI has been shown to be an effective and cheap treatment to overcome the debilitating consequences of LARS^12^, ^13^, ^16^, ^17^, ^18^, ^19^. It was therefore the aim of this trial to examine the effect of prophylactic TAI after closure of the protective stoma in order to prevent the onset of LARS in the early postoperative period. Patients who underwent 1000 ml of irrigation every 24 h as instructed by a dedicated therapist had significantly fewer bowel movements and visits to the toilet during the day and at night, as well as better LARS and Wexner scores within 1 month of ileostomy closure, which persisted until 3 months of follow‐up.

Defaecation episodes (and visits to the toilet) at night were reduced to almost none after 1 month. Although multiple bowel movements during daytime must be regarded as a significant burden in daily life, being unable to have a single night of undisturbed sleep (owing to multiple unproductive defaecation episodes with or without episodes of incontinence) seems likely to have an additional detrimental impact on QoL. Evaluation of QoL by means of the SF‐36® questionnaire nevertheless failed to show any differences between the groups, reflecting the small sample size in this study, as well as the fact that this generic instrument does not cover many of the specific aspects of LARS.

The present results mirror outcomes in other studies. An observational study^18^ found a significant decrease in the number of bowel movements from a median of 7 at baseline to 1 after 6 months, with a change in median LARS score from 35·1 to 12·2. In a recent study^19^ using an antegrade irrigation system via a percutaneous endoscopic caecostomy, of 25 patients considered candidates for permanent colostomy because of severe LARS and/or incontinence, only three had to proceed to the formation of a permanent stoma.

After ultralow rectal resection, problems associated with LARS can start within a few days of protective ileostomy closure^6^, ^7^, ^8^, ^9^. Although it has been advocated that formation of a neoreservoir might reduce this problem^20^, pouch formation after ultralow resection is often not feasible technically, nor does it seem to provide a solution to all the problems encountered in LARS^21^.

Although the results of the present trial indicated that TAI should be offered to patients after ultralow rectal resection before the onset of LARS, questions remain regarding the optimal volume of water required for TAI, time interval between irrigations, long‐term safety and whether TAI should be considered a lifelong therapy.

In the study^12^ that served as the basis for the protocol used in the present trial, the median volume of water used for irrigation was 900 (500–1500) ml every 24 h. Martellucci and co‐workers^18^ used a median volume of only 450 (300–1000) ml three to four times per week, although the authors reported six dropouts among 33 patients, some owing to dissatisfaction with the treatment.

A risk of rectal perforation during TAI should be acknowledged. Incidents of perforation have been reported when TAI was used for other indications, including anal atresia, and neurogenic sources of incontinence and constipation^22^. A global audit^22^ of the risk of perforation during TAI recorded by the European Community and the US Food and Drug Association estimated an average risk of one in 167 000 for bowel perforation during TAI. In comparison, the incidence of perforation during colonoscopy has been reported to be in the range of one in 1000 procedures. Evaluation of healing of the rectal anastomosis by endoscopy should be mandatory in every patient before TAI is initiated, and training and counselling of all patients by an experienced therapist is needed. Recent observations of a French group^23^ also showed that professional training was a major key factor in the success of TAI.

It is widely accepted that LARS symptoms can improve spontaneously over time, raising the question of the expected total duration of TAI treatment. As most patients treated by TAI for LARS have been chronic sufferers whose symptoms have been refractory to other treatments^12^, ^13^, ^18^, ^19^, this question is difficult to answer. In the study by Martellucci and co‐workers^18^, TAI was suspended after 6 months of follow‐up and substituted by regular enemas. The authors stated that 85 per cent of the patients returned to a TAI protocol owing to rapid recurrence of LARS symptoms.

Although the present trial focused on the immediate effect of TAI after ileostomy closure, the protocol offers patients the opportunity to cross over into the other group after the 3‐month follow‐up has been completed. Patients who continue in the TAI group will have the possibility to reduce irrigation volumes by 100 ml per week to see how this influences symptoms and QoL. These effects will be evaluated after all patients have completed 1 year of follow‐up after ileostomy closure.
